# Feasibility of extended cycles of neoadjuvant chemotherapy in patients with advanced ovarian cancer in terms of prognosis and surgical outcomes

**DOI:** 10.1371/journal.pone.0284753

**Published:** 2023-04-21

**Authors:** Nam Kyeong Kim, Dong Hoon Suh, Kidong Kim, Yong Beom Kim, Jae Hong No

**Affiliations:** 1 Department of Obstetrics and Gynecology, Seoul National University Bundang Hospital, Seongnam, Korea; 2 Department of Obstetrics and Gynecology, Seoul National University College of Medicine, Seoul, Korea; Sapienza University of Rome: Universita degli Studi di Roma La Sapienza, ITALY

## Abstract

**Objective:**

We aimed to identify the effect of an extended number of neoadjuvant chemotherapy (NAC) cycles on prognosis and surgical morbidity after interval debulking surgery (IDS) in patients with newly diagnosed advanced ovarian cancer.

**Methods:**

Medical records of patients with advanced ovarian cancer treated with NAC and having undergone IDS were retrospectively reviewed. Clinicopathological factors were compared between two groups: conventional (≤4 cycles) and extended (≥5 cycles) NAC groups. Kaplan–Meier analysis was performed to evaluate progression-free survival (PFS) and overall survival (OS).

**Results:**

A total of 156 patients were included, 112 patients in the conventional group and 44 patients in the extended NAC group. The extended NAC group had a significantly higher frequency of cancer antigen (CA)-125 normalization after NAC (59.1% vs. 33.9%, *P* = 0.004), a lower rate of bowel surgery (18.2% vs. 34.8%, *P* = 0.042), and a lower rate of transfusion during or after IDS (36.4% vs. 59.8%, *P* = 0.008) as compared to the conventional group. The complete cytoreduction rate after IDS was similar between the groups. In multivariate Cox regression analysis for PFS, radiologically stable and progressive disease after NAC (Hazard ratio [HR], 1.983; 95% Confidence interval [CI], 1.141–3.446; *P* = 0.015) and gross residual tumor after IDS (HR, 2.054; 95% CI, 1.414–2.983; *P* < 0.001) were independent risk factors for poor PFS. However, extended NAC cycles were not significantly associated with poor PFS. The median PFS was 19.5 and 16.9 months (*P* = 0.830), and the 5-year OS was 71.4 and 63.2% (*P* = 0.677) in the conventional and extended NAC groups, respectively.

**Conclusion:**

Our study showed that extended NAC cycles were not inferior to conventional NAC cycles in terms of survival in patients with advanced ovarian cancer and reduced surgical morbidity such as bowel surgery and transfusion during or after IDS.

## Introduction

Among gynecological cancers, ovarian cancer is known to have poor prognosis. The 5-year survival rate for advanced ovarian cancer is less than 50% despite the development of therapeutic agents [1]. In patients with ovarian cancer, 70% are initially diagnosed at an advanced stage [2]. Primary debulking surgery (PDS) followed by platinum and paclitaxel chemotherapy is considered the basis of treatment for advanced ovarian cancer. Residual tumor after PDS is an important prognostic factor for progression-free survival (PFS) and overall survival (OS) in patients with stage IIB or higher ovarian cancer, and the goal of PDS should be complete resection of the tumor [3]. A study on the extent of PDS and postoperative complications in patients with stage III or higher ovarian cancer reported that 32% of the patients underwent extensive pelvic surgery, including bowel resection or extensive upper abdominal surgery, such as splenectomy, pancreatectomy, and hepatectomy [4]. Extensive surgery was significantly associated with postoperative complications [4–6].

Several recent, large randomized trials comparing PDS and interval debulking surgery (IDS) after three to four cycles of neoadjuvant chemotherapy (NAC) in patients with advanced ovarian cancer have shown that IDS following NAC is not inferior to PDS in terms of survival [7–10]. Based on these results, National Comprehensive Cancer Network (NCCN) recommends NAC followed by IDS as a standard treatment option for patients having a low probability of optimal cytoreduction after PDS or who are unable to undergo surgery because of medical problems [11]. A meta-analysis reported that IDS after NAC was associated with superior optimal cytoreduction, lower perioperative morbidity, post-surgical mortality, and better quality of life as compared to PDS [12].

A retrospective analysis reported that six cycles of neoadjuvant carboplatin and paclitaxel chemotherapy were safe and effective and did not increase peri- or postoperative complications in advanced ovarian cancer [13]. According to the NCCN guidelines, three to four cycles of NAC are preferred, but IDS may be performed after four to six cycles of NAC based on the clinical judgement of gynecologic oncologists [11]. Based on the findings of various studies, more than four cycles of NAC are considered feasible [11, 13]. However, previous studies on the number of NAC cycles did not focus on surgical morbidity, including the extent of IDS, and the results on prognosis were inconsistent. Therefore, the present study aimed to identify the effect of an extended number of NAC cycles on prognosis and surgical morbidity after IDS in patients with newly diagnosed, advanced ovarian cancer.

## Materials and methods

### Patients

A retrospective analysis was performed for all patients with newly diagnosed, advanced ovarian cancer who were treated with NAC and underwent IDS at Seoul National University Bundang Hospital (SNUBH) between June 2003 and December 2020. The 2014 International Federation of Gynecology and Obstetrics (FIGO) staging system was used for the staging of ovarian cancer. Patients diagnosed with FIGO stage IIIB or higher epithelial ovarian, fallopian, or primary peritoneal cancer and who underwent IDS after NAC at SNUBH were included in this study. The exclusion criteria were as follows: 1) histological types other than epithelial ovarian cancer; 2) concurrent other cancer or double primary cancer that could affect prognosis; and 3) patients with no follow-up after IDS in SNUBH or who received treatment after IDS in another hospital. This study was approved by the Institutional Review Board of our institute (B-2301-807-116), and the need for informed consent was waived because of the retrospective nature of the study.

### Definitions

The study population was classified into two groups according to the number of NAC cycles: the conventional (≤4 cycles) and the extended (≥5 cycles) NAC group. Because the NCCN guidelines and well-known Randomized Controlled Trials (RCTs) comparing NAC and PDS recommend three to four cycles of NAC, the cutoff number of conventional NAC was set to four in our study [7–11]. Information regarding age at diagnosis, FIGO stage, histology, grade, number of NAC cycles, NAC regimen, radiologic response after NAC, pre/post NAC cancer antigen (CA)-125, and adjuvant chemotherapy was collected. Data on IDS, including the status of residual tumor, operation time, red blood cell (RBC) transfusion during operation or immediately post-operation, and the extent of IDS, were collected. Bowel surgery such as lower anterior resection, colectomy, or small bowel resection and anastomosis, and upper abdominal surgery such as splenectomy, pancreatectomy, and hepatectomy were investigated as the extent of IDS. Ovarian cancer was initially diagnosed using cytology or biopsy prior to NAC. Radiologic response to NAC was evaluated using the Response Evaluation Criteria in Solid Tumors 1.1. Cancer recurrence was defined as radiological confirmation by computerized tomography, magnetic resonance imaging, or positron emission tomography. CA-125 normalization was defined at less than 35 U/mL after NAC.

### Statistical analysis

Pearson’s χ ^2^ test, Fisher’s exact test, or Student’s t-test were used to compare characteristics according to the number of NAC cycles and recurrence of cancer. Univariate and multivariate logistic regression analyses were used to identify factors affecting the recurrence of ovarian cancer, including the number of NAC cycles. Kaplan–Meier analysis was performed to evaluate PFS and OS. The values in the tables are presented as hazard ratios (HR), 95% confidence intervals (CI), and p-values. Statistical analyses were performed using IBM SPSS Statistics for Windows (version 25.0; IBM Corp., Armonk, NY, USA). A *P*-value < 0.05 was considered statistically significant.

## Results

A total of 156 patients who met the inclusion criteria were enrolled in this study. The clinical characteristics of the study population are presented in Table 1. The mean age of patients at the time of cancer diagnosis was 59.1 years. The most common histological type was serous adenocarcinoma (129 cases, 82.7%). The mean value of CA-125 before treatment was 3090.7 U/mL, and that after NAC was 228.5 U/mL. One hundred thirty-seven (87.8%) patients evaluated radiologically showed a partial response to NAC treatment. The median number of cycles of NAC was three (range, 2–8) and the median number of total chemotherapy cycles, including NAC and adjuvant chemotherapy, was nine (range, 5–24). Among the study population, 148 patients (94.9%) received more than two cycles of adjuvant chemotherapy, and 38 patients (24.4%) received maintenance treatment. A comparison of clinical factors between the conventional and extended NAC groups is shown in Table 1. Age at diagnosis, pretreatment CA-125 level, radiologic response after NAC, histology, and maintenance treatment were similar between the conventional and extended NAC groups. The frequency of FIGO stage IV tended to be higher in the extended as compared to the conventional NAC group, but there was no significant difference (70.5% vs. 55.4%, *P* = 0.084). This could suggest that the extended NAC group included patients with a higher tumor burden than the conventional NAC group. The mean CA-125 level after NAC was significantly lower in the extended NAC group than that in the conventional NAC group (55.0 U/mL vs. 296.7 U/mL, *P* < 0.001). Additionally, the rate of CA-125 normalization after NAC was significantly higher in the extended than in the conventional NAC group (59.1% vs. 33.9%, *P* = 0.004). The frequency of receiving two or more cycles of adjuvant chemotherapy was significantly higher in the conventional than in the extended NAC group (99.1% vs. 84.1%, p = 0.001). The total number of chemotherapies, including NAC and adjuvant chemotherapy, showed no significant difference between the two groups (conventional vs. extended NAC group; mean ± standard deviation; 8.88 ± 1.71 vs. 9.98 ± 3.47, p = 0.051), although there was a significant difference in the number of adjuvant chemotherapies (5.82 ± 1.70 vs. 3.95 ± 3.27; *P* = 0.001).

**Table 1:** Clinical characteristics between the conventional and extended NAC groups.

Variables	Overall population (n = 156)	Conventional NAC group (n = 112)	Extended NAC group (n = 44)	P-value
Age at diagnosis	59.1 ± 10.5	59.3 ± 10.1	58.6 ± 11.6	0.708
Pretreatment CA-125 (U/mL)	3090.7 ± 6059.6	3083.7 ± 6510.9	3108.4 ± 4788.5	0.982
FIGO stage				0.084
IIIB, IIIC	63 (40.4)	50 (44.6)	13 (29.5)	
IVA, IVB	93 (59.6)	62 (55.4)	31 (70.5)	
CA-125 after NAC (U/mL)	228.5 ± 570.3	296.7 ± 660.0	55.0 ± 70.5	<0.001
≤ 35 U/mL	64 (41.0)	38 (33.9)	26 (59.1)	0.004
> 35 U/mL	92 (59.0)	74 (66.1)	18 (40.9)	
Radiologic response after NAC				0.591
PR	137 (87.8)	97 (86.6)	40 (90.9)	
SD, PD	19 (12.2)	15 (13.4)	4 (9.1)	
Histology				0.515
Non-serous^a^	27 (17.3)	18 (16.1)	9 (20.5)	
Serous	129 (82.7)	94 (83.9)	35 (79.5)	
Number of chemotherapy cycles				
NAC	3.9 ± 1.4	3.06 ± 0.31	6.02 ± 0.66	<0.001
Adjuvant chemotherapy	5.3 ± 2.4	5.82 ± 1.70	3.95 ± 3.27	0.001
Total chemotherapy	9.2 ± 2.4	8.88 ± 1.71	9.98 ± 3.47	0.051
Maintenance treatment				0.123
No	118 (75.9)	81 (72.3)	37 (84.1)	
Yes	38 (24.4)	31 (27.7)	7 (15.9)	
Types of maintenance treatment				0.812
Bevacizumab	15/38 (39.5)	13/31 (41.9)	2/7 (28.6)	
PAPR inhibitor	8/38 (21.1)	6/31 (19.4)	2/7 (28.6)	
Others^b^	15/38 (39.5)	12/31 (38.7)	3/7 (42.8)	

Values are presented as number of patients (%) or mean ± standard deviation.

FIGO, International Federation of Gynecology and Obstetrics; NAC, neoadjuvant chemotherapy; PR, Partial Response; SD, Stable Disease; PD, Progressive Disease; PARP, Poly (ADP-ribose) polymerase.

^a^ Non-serous histology including four endometrioid, five clear cell, seven mucinous, three carcinosarcoma, eight others.

^b^ Others including two monthly paclitaxel, one oral etoposide, and 12 clinical trials (7 ATHENA, 4 MK-7339, 1 DUO-O).

Table 2 shows surgical outcomes and prognosis in the study population and the two groups. In the overall study population, mean operation time was 248.5 min, and RBC transfusions were performed in 83 patients (53.2%), either during or immediately after IDS. During IDS, 47 patients (30.1%) underwent bowel surgery and 17 patients (10.9%) underwent upper abdominal surgery. The complete cytoreduction rate (no residual tumor) was 57.7% (90 patients). In the extended NAC group, the mean operation time was shorter than that in the conventional NAC group by approximately 30 min; however, the difference was not statistically significant (299.4 ± 142.8 min in the conventional vs. 265.1 ± 118.9 min in the extended NAC group; *P* = 0.159). The frequency of bowel surgery and RBC transfusion rate during or immediately after IDS were significantly lower in the extended as compared to the conventional NAC group (rate of bowel surgery, 18.2% vs. 34.8%; *P* = 0.042, rate of RBC transfusion, 36.4% vs. 59.8%; *P* = 0.008). The frequency of upper abdominal surgery also tended to be lower in the extended NAC group without significant difference (4.5% vs. 13.4%, *P* = 0.090). Both groups had a similar complete cytoreduction rate (conventional vs. extended NAC group; 55.0% vs. 65.9%, *P* = 0.213). The recurrence rate between the two groups was not significantly different (conventional vs. extended NAC group; 83.0% vs. 77.3%, *P* = 0.405).

**Table 2:** Surgical outcomes and prognosis between the conventional and extended NAC groups.

	Overall population (n = 156)	Conventional NAC group (n = 112)	Extended NAC group (n = 44)	P-value
Operation time (minute)	289.7 ± 137.0	299.4 ± 142.8	265.1 ± 118.9	0.159
RBC transfusion during or immediately after IDS	83 (53.2)	67 (59.8)	16 (36.4)	0.008
Extent of IDS				
Including bowel surgery	47 (30.1)	39 (34.8)	8 (18.2)	0.042
Including upper abdomen surgery	17 (10.9)	15(13.4)	2 (4.5)	0.090
Complete cytoreduction after IDS ^a^				0.213
No	65 (41.9)	50 (45.0)	15 (34.1)	
Yes	90 (58.1)	61 (55.0)	29 (65.9)	
Recur				0.405
No	29 (18.6)	19 (17.0)	10 (22.7)	
Yes	127 (81.4)	93 (83.0)	34 (77.3)	

Values are presented as number of patents (%) or mean ± standard deviation.

NAC, neoadjuvant chemotherapy; RBC, red blood cells; IDS, interval debulking surgery.

^a^ One data was missing.

The results of the univariate and multivariate logistic regression analyses of the factors affecting the recurrence of ovarian cancer are shown in Table 3. In the univariate analysis, CA-125 > 35 U/mL after NAC and residual disease status were associated with cancer recurrence. After adjusting for other relevant factors, including FIGO stage and histological type, the results of the multivariate logistic regression analysis showed that CA-125 > 35 U/mL after NAC (HR, 3.729; 95% CI, 1.503–9.254; *P* = 0.005) and residual tumor after IDS (HR, 2.774; 95% CI, 1.095–7.028; *P* = 0.031) were independent prognostic factors affecting the recurrence of ovarian cancer, while NAC with extended cycles was not a significant factor. Multivariate Cox regression analysis for PFS (Table 4) showed that radiologically stable disease (SD) and progressive disease (PD) after NAC (HR, 1.983; 95% CI, 1.141–3.446; *P* = 0.015) and gross residual tumor after IDS (HR, 2.054; 95% CI, 1.414–2.983; *P <* 0.001) were independent risk factors for poor PFS. However, NAC with extended cycles was not a significant risk factor for poor PFS.

**Table 3:** Univariate and multivariate logistic regression analysis of factors affecting recurrence of ovarian cancer.

	Patients (%)	Univariate			Multivariate^a^		
		HR	95% CI	P-value	HR	95% CI	P-value
Age at diagnosis							
≤ 50	35 (22.4)	1					
> 50	121 (77.6)	1.125	0.436–2.904	0.808			
Pretreatment CA-125							
≤ 3090 U/mL	114 (73.1)	1					
> 3090 U/mL	42 (26.9)	1.196	0.469–3.047	0.708			
FIGO stage							
IIIB, IIIC	63 (40.4)	1			1		
IVA, IVB	93 (59.6)	1.486	0.660–3.344	0.339	1.875	0.779–4.513	0.161
Number of NAC cycles							
> 4 cycles	44 (28.2)	1					
≤ 4 cycles	112 (71.8)	1.440	0.609–3.404	0.407			
CA-125 after NAC							
≤ 35 U/mL	64 (41.0)	1			1		
> 35 U/mL	92 (59.0)	3.462	1.483–8.082	0.004	3.729	1.503–9.254	0.005
Radiologic response after NAC							
PR	137 (87.8)	1					
SD, PD	19 (12.2)	2.086	0.454–9.582	0.344			
Residual disease^b^							
No	90 (58.1)	1			1		
Yes	65 (41.9)	2.681	1.068–6.726	0.036	2.774	1.095–7.028	0.031
Histology							
Non-serous	27 (17.3)	1			1		
Serous	129 (82.7)	1.702	0.642–4.515	0.285	2.786	0.933–8.326	0.067

FIGO, International Federation of Gynecology and Obstetrics; NAC, neoadjuvant chemotherapy; PR, partial response; SD, stable disease; PD, progressive disease.

^a^ Enter method.

^b^ One data was missing.

**Table 4:** Univariate and multivariate Cox regression analysis for progression-free survival.

	Patients (%)	Univariate			Multivariate^a^		
		HR	95% CI	p-value	HR	95% CI	p-value
Age at diagnosis							
≤ 50	35 (22.4)	1			1		
> 50	121 (77.6)	1.316	0.864–2.005	0.200	1.506	0.978–2.319	0.063
Pretreatment CA-125							
≤ 3090 U/mL	114 (73.1)	1					
> 3090 U/mL	42 (26.9)	0.989	0.670–1.461	0.957			
FIGO stage							
IIIB, IIIC	63 (40.4)	1			1		
IVA, IVB	93 (59.6)	1.302	0.909–1.866	0.150	1.422	0.988–2.047	0.058
Number of NAC cycles							
> 4 cycles	44 (28.2)	1					
≤ 4 cycles	112 (71.8)	1.044	0.705–1.547	0.830			
CA-125 after NAC							
≤ 35 U/mL	64 (41.0)	1			1		
> 35 U/mL	92 (59.0)	1.613	1.119–2.326	0.010	1.374	0.938–2.012	0.103
Radiologic response after NAC							
PR	137 (87.8)	1			1		
SD, PD	19 (12.2)	2.003	1.199–3.346	0.008	1.983	1.141–3.446	0.015
Residual disease^b^							
No	90 (58.1)	1			1		
Yes	65 (41.9)	2.144	1.503–3.059	<0.001	2.054	1.414–2.983	<0.001
Histology							
Non-serous	27 (17.3)	1					
Serous	129 (82.7)	1.138	0.705–1.836	0.596			

FIGO, International Federation of Gynecology and Obstetrics; NAC, neoadjuvant chemotherapy; PR, partial response; SD, stable disease; PD, progressive disease.

^a^ Enter method.

^b^ One data was missing.

**Fig 1:**
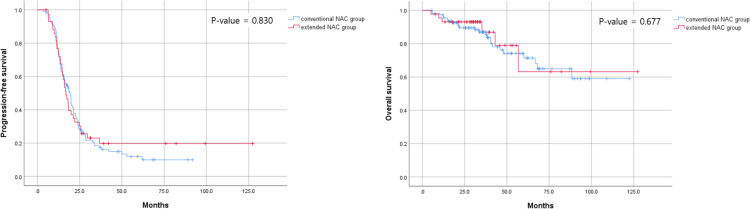
Kaplan-Meier curve for survival between the conventional and extended NAC groups. (A) progression-free survival and (B) overall survival. NAC, neoadjuvant chemotherapy.

The results of the Kaplan–Meier analysis show no significant differences in both PFS (Fig 1A) and OS (Fig 1B) between the conventional and extended NAC groups. The median PFS was 19.5 months (95% CI, 16.5–22.4) in the conventional and 16.9 months (95% CI, 14.1–19.7) in the extended NAC group. Furthermore, the 5-year OS was 71.4% in the conventional and 63.2% in the extended NAC group, with no significant difference between the two groups (*P* = 0.677).

## Discussion

### Main findings of the study

This study showed that extended NAC cycles resulted in non-inferior survival and reduced surgical morbidity, such as bowel surgery and transfusion, compared with conventional NAC cycles. Moreover, NAC with extended cycles was not a significant risk factor for the recurrence of ovarian cancer and poor PFS. There was no significant difference in survival between the two groups, which might be related to a similar complete cytoreduction rate and total number of chemotherapy cycles in both groups.

### Implications and comparison with the literature

Several RCTs have demonstrated that three or four cycles of NAC followed by IDS are not inferior to PDS in terms of survival outcomes in advanced ovarian cancer [7–10]. In our study, the frequency of FIGO stage IV (59.6% vs. the RCTs 9.2–30.9%) and the CA-125 level at initial diagnosis (mean CA-125, 3091 U/mL vs. the RCTs 2100 U/mL) were higher than those reported in previous RCTs on NAC. Thus, more high-risk patients with a higher tumor burden may have been included in this study. According to our findings, extended NAC cycles in these high-risk patients did not lead to worse survival outcomes as compared to conventional NAC cycles. A previous study using propensity score matching analysis showed that receiving more than four cycles of NAC was not detrimental in terms of OS and PFS in advanced ovarian cancer. There were no significant differences in OS (2-year OS: 82.4% for NAC ≤4 cycles cohort vs. 77.1% for NAC >4 cycles cohort, *P* = 0.109) and PFS (2-year PFS: 29.7% for NAC ≤4 cycles cohort vs. 20.0% for NAC >4 cycles cohort, *P* = 0.875) [14]. These survival outcomes were in close agreement with our results. However, unlike our study, the authors did not investigate the surgical morbidity during or after IDS. Addition of bevacizumab to first-line chemotherapy and bevacizumab maintenance therapy improved survival outcomes in high-risk ovarian cancer patients [15]. Clinical trials of adding bevacizumab to NAC did not show improvement of the complete cytoreduction rate and PFS, but improvement of surgical operability without increasing toxicity. The feasibility and safety of adding bevacizumab to NAC have been reported, but evidence is lacking to draw definitive conclusions and to select appropriate patients [15–17]. In our study, platinum-based chemotherapy with bevacizumab as NAC regimen was used in a small number of patients in both the conventional and extended NAC groups (6.3% (7/112) vs. 7.1% (3/42), *P* > 0.999). Therefore, the effect of bevacizumab on survival in this study could be considered small and was unlikely to differ between the two groups.

Some studies have shown that extended NAC cycles are associated with unfavorable survival outcomes. A previous retrospective study on preoperative NAC cycles and survival reported that receiving five or more NAC cycles was associated with worse PFS and OS, and the negative prognosis was not relieved by complete gross resection [18]. However, that study had a short follow-up duration of <2 years and did not consider the total number of chemotherapy cycles. This negative effect on prognosis may be explained by the hypothesis that NAC is associated with platinum resistance, although no definitive conclusion can be drawn from the current evidence [19]. However, one retrospective study found that patients who received four or more cycles of NAC did not have an increased risk of platinum resistance or worsened survival [20]. In the current study, there was no significant difference in platinum-resistant recurrence rates between the conventional and extended NAC groups (33.7% (31/92) vs. 42.9% (12/28), *P* = 0.376).

A recent study of systematic lymphadenectomy in debulking surgery for advanced ovarian cancer did not report better survival outcomes but higher complication and mortality rates associated with lymphadenectomy [21]. In our study, there was no significant difference in the frequency of pelvic and paraaortic lymphadenectomy between the conventional and the extended NAC groups (43.8% vs. 38.6%, *P* = 0.561). Cox regression analysis showed that systematic lymphadenectomy was not associated with better PFS. Our findings were consistent with those of the previous study. However, the current study has limitations related to various surgical factors and heterogeneous lymphadenectomy procedures due to the retrospective nature of the study.

### Future directions

Platinum-resistant or platinum-refractory advanced ovarian cancer has poorer prognosis than platinum-sensitive ovarian cancer and has recently been suggested as inherently resistant to chemotherapy associated with the immune system and tumor microenvironment [22]. Although our study population was chemotherapy naïve, the radiologic response after NAC was SD and PD in 14 (9.0%) and five (3.2%) patients, respectively. These patients were significantly associated with poor PFS. This resistance to NAC may also be related to the immune system or tumor microenvironment. Further studies of markers related to the immune system or tumor microenvironment are needed to initially screen for patients who do not respond to NAC. Research on immunotherapies that can render NAC-resistant tumors more sensitive to chemotherapy by changing the tumor microenvironment and immune system are needed as well.

Frailty may also affect the clinician’s decision to extend the number of NAC cycles and the prognosis of ovarian cancer according to the number of NAC cycles. NAC can be considered a first-line treatment for elderly women who have medical problems and are expected to have serious morbidity or mortality after debulking surgery. Frailty is evaluated systematically through frailty assessment and cannot be simply judged by age. One review article about frailty assessments reported that frail patients had a lower disease-free and overall survival and more adverse postoperative outcomes than non-frail patients [23]. To more accurately evaluate the effect of the number of NAC cycles on survival and postoperative outcomes and select appropriate patients requiring extended cycles of NAC, it is necessary to classify patients via frailty assessment and perform further subgroup analysis. In our study, the mean age at diagnosis was similar between the conventional and extended NAC groups (59.3 ± 10.1 vs. 58.6 ± 11.6, *P* = 0.708). However, the current study was limited in assessing frailty through review of medical records because of the retrospective nature of the study.

## Strengths and limitations of the study

This study focused on survival and surgical morbidity based on the number of NAC cycles. However, the study has several limitations. First, the number of NAC cycles and timing of IDS were determined according to the clinical judgement of various gynecological or hematological oncologists at our institution. Most patients received extended NAC cycles to further reduce the tumor burden; however, in some cases, the reasons for extended NAC cycles were unclear when reviewed retrospectively. Second, we collected data between 2003 and 2020, and data before and after the introduction of ovarian cancer maintenance treatment were mixed in this study. Since 2015, maintenance treatment such as bevacizumab or poly ADP ribose polymerase inhibitors have been introduced for the treatment of ovarian cancer, resulting in improved survival [24, 25]. Moreover, as this study was conducted retrospectively, chemotherapy-related toxicity and quality of life according to the number of NAC cycles were not investigated.

## Conclusions

In conclusion, extended NAC cycles showed non-inferior survival outcomes compared to conventional NAC cycles in patients with advanced ovarian cancer and could reduce surgical morbidity, such as bowel surgery and transfusion during or after IDS. For patients with unresectable, advanced ovarian cancer and those whose surgical extent is still expected to be too extensive after conventional NAC, extended NAC followed by IDS may be considered as a primary treatment option to reduce bowel and upper abdominal surgery. Further randomized studies are needed to identify optimal criteria for patients requiring extended NAC cycles and to determine the optimal number of NAC cycles considering both survival and quality of life.
